# Inhibition of PKM2 Enhances Sensitivity of Olaparib to Ovarian Cancer Cells and Induces DNA Damage

**DOI:** 10.7150/ijbs.62947

**Published:** 2022-01-24

**Authors:** Sichun Zhou, Duo Li, Di Xiao, Tianyu Wu, Xin Hu, Yu Zhang, Jun Deng, Jing Long, Simeng Xu, Jingtao Wu, Gaofeng Li, Mei Peng, Xiaoping Yang

**Affiliations:** 1 Key Laboratory of Study and Discovery of Small Targeted Molecules of Hunan Province, Department of Pharmacy, School of Medicine, Hunan Normal University, Changsha, 410013, China; Key Laboratory of Molecular Pharmacology and Drug Evaluation, Ministry of Education, Yantai University, Yantai, 264005, China; 2 Department of Oncology, The Affiliated Zhuzhou Hospital of Xiangya School of Medicine, Central South University, Zhuzhou, 412007, China; Obstetrics and Gynecology Department, Xiangya Hospital, Central South University, Changsha, 410008, China; Departments of Pharmacy, Xiangya Hospital, Central South University, Changsha, 410008, China

**Keywords:** ovarian cancer, olaparib, PKM2, DNA damage, homologous recombination

## Abstract

Poly (ADP-ribose) polymerase inhibitors (PARPi) have showed clinical benefit as maintenance therapy in advanced ovarian cancer by impairing the homologous recombination (HR) pathway. Pyruvate kinase M2 (PKM2), the significant cancer metabolic biomarker, integrates with DNA damage to directly promote HR. We aimed to investigate the role and molecular mechanism of PKM2 downregulation on sensitization of ovarian cancer cells to PARPi. Inhibitory effects in vitro were assessed by cell viability, clone formation, transwell assay, and flow cytometry. Downregulation of PKM2 by siRNA or small molecular inhibitor shikonin (Sk) enhanced anti-tumour activity of olaparib (Ola) in ovarian cancer cells. Silencing PKM2 or Sk synergized with Ola and reduced cell growth, colony formation and migration, and induced apoptosis. Western blot and immunofluorescence demonstrated that inhibition of PKM2 amplified Ola-induced γH2AX and phospho-ATM (p-ATM) activation and interfered with BRCA1 accumulation in the nucleus. A xenograft animal model demonstrated in vivo antitumor combination effect of Sk and Ola. Furthermore, Western blot and immunofluorenscent analyses of tissue samples revealed that treatment of Sk increased DNA damage, reduced expression of BRCA1 and PKM2. Therefore, this study identified that PKM2 downregulation is a novel therapeutic strategy to enhance Ola effectiveness in treating ovarian cancer.

## Introduction

Ovarian cancer is a common and deadly gynaecologic malignancy 1. Due to lack of symptoms at early stages and proper screening methods for precancerous lesions, many ovarian cancer cases are detected until middle and advanced stages 2. The first-line chemotherapeutic regimen for late stage ovarian cancer is paclitaxel in combination to carboplatin 3-5. However, 70% of patients with middle and advanced stages receiving surgery and chemotherapy survived less than five years after diagnosis 4.

Tumor-targeted therapy has achieved great success and gradually become a first-line treatment strategy due to its specificity, effectiveness and relatively low toxicity 6-8. Several efficient targeted administrations exist for ovarian cancer treatments of which Poly (ADP-ribose) polymerase inhibitors (PARPi) are the prevalent and promising drugs 9-12. PARPi have shown clear advantage in BRCA-mutated ovarian cancer by inhibiting base excision repair (BER) and advancing DNA single-strand breaks (SSB) into double-strand breaks (DSB) by blocking the homologous recombination (HR) repair pathway leading to genomic instability 13, 14. BRCA1 and BRCA2 are two critical tumor suppressor genes for the repair of DNA double-strand breaks through HR pathway 15. Several studies noted that the BRCA1 expression is negatively associated with progression-free survival and overall survival in epithelial ovarian cancer 16. When both PARP and BRCA1/2 were inhibited, cell mortality significantly increased, which is also referred to as “synthetic lethality” 14. Burgess *et al* showed that the IC_50_ value of BRCA1 wildtype ovarian cancer cells is 10-fold larger than that of mutant ovarian cancer cells 17. Thus, PARPi are far less effective as monotherapy for BRCA wildtype ovarian cancer patients.

According to the gene map of ovarian cancer patients, approximately 59% of all epithelial ovarian cancers and 50% of all high-grade advanced ovarian carcinomas retain BRCA1/2 expression 18. Many studies showed that increasing DNA damage of cancer cell and inhibiting HR repair pathways are the major methods to improve the treatment efficiency of olaparib (Ola) in BRCA1 wild-type ovarian cancer 19-21. Pyruvate kinase (PK) catalyzes the final irreversible reaction of glycolysis, which accelerates the conversion of phosphoenolpyruvate to pyruvate and regulates the flux of acetyl-CoA that can enter the tricarboxylic acid-assisted cycle 22-24. M1, M2, R and L are four subtypes of PK, of which pyruvate kinase M2 (PKM2) is most commonly overexpressed in tumors 25. Particularly, it has been found that ovarian tumors show metabolic adaptation to aerobic glycolysis that allows them to maintain an increased proliferative capacity and survive under anchorage dependent conditions 26. Shikonin (Sk) is one of the most potent and selective inhibitors of PKM2 27-30, with a 10-20-fold higher selectivity for PKM2 vs PKM1 and PKL 31. This makes Sk a useful tool to study the downregulation of PKM2 induces DNA damage by disrupting HR in ovarian cancer cells.

The latest studies indicated that targeting PKM2 for inhibition could effectively increases sensitivity to chemotherapy and radiotherapy 32, 33, and it may be related to PKM2 could enter the nucleus via the phosphorylation integrates with the DNA damage response to directly promote DNA double-strand break (DSB) repair 34. Our group discovered that downregulation of PKM2 sensitized bladder cancer cells to the cytotoxic effects of pirarubicin (a DNA damaging agent) 35. Therefore, PKM2-targeting strategies as means to not only disrupt cancer metabolism but also increase sensitivity to genotoxic therapies. However, whether PKM2 plays a significant role in the regulation of Ola sensitivity in ovarian cancer cells remains to be elucidated. Formulating treatment strategies for BRCA wild type ovarian cancer patients needs to be further investigated. In the current study, we show that inhibition of PKM2 by siRNA or small molecular inhibitor (Sk) induced DNA damage by activating ATM/γH2AX and disrupts BRCA1 expression. *In vivo* and *in vitro* experiments, we have verified that inhibiting PKM2 can effectively increase the anti-tumor effect of Ola. This novel finding indicates that coordination between PKM2 and DNA double-strand break repair may be an efficient approach to increase the sensitivity of Ola.

## Materials and methods

### Chemicals and antibodies

Ola and Sk were purchased from Targetmol (Shanghai, China). Both Ola and Sk were dissolved in dimethyl sulfoxide (DMSO), dissolution with a 20 mM stock solution. Primary antibodies against β-Actin (#8457), PKM2 (#4053), γH2AX (phosphoSer139; #9718) were provided by Cell Signaling Technology (CST, Boston, USA). p-ATM (phosphoSer1981; ab81292) was provided by Abcam (Cambridge, UK). BRCA1 (22362-1-AP) was provided by Proteintech (Wuhan, China). The secondary antibodies anti-rabbit IgG (#7074) and anti-mouse IgG (#7076) were provided by CST. Alexa Fluor 488-conjugated Affinipure goat anti-rabbit (SA00006-2) and Cy3-conjugated Affinipure goat anti-mouse (SA00009-1) were provided by Proteintech.

### Cell culture

Human ovarian cancer A2780, SKOV3 and OVCAR3 cell lines provided by Dr. Y Zhang were cultured in RPMI-1640 medium supplemented with 10% fetal bovine serum and 1% penicillin-streptomycin (Invitrogen, USA) at 37℃ in a humidified atmosphere with 5% CO_2_.

### Human ovarian carcinoma tissues

The study protocol was approved by the Ethics Committee of School of Medicine, Hunan Normal University (IRB No: 2018027A). All patients received the informed consent forms and agreed to participate in the study. Immunohistochemistry assay was performed to measure the protein levels of PKM2 in ovarian carcinoma tissues (n=20) and adjacent ovarian normal tissues (n=10). Briefly, the formalin-fixed and paraffin-embedded 5-μm tissue sections were dewaxed and rehydrated. To unmask the antigen and inactivolate endogenous peroxidase, the rehydrated sections were treated with Tris-EDTA buffer solution (it contains 10mM Tris-Hcl and 1mM EDTA). Sections were then heated to boil in a pressure cooker for 3 min, naturally cold cut, washed 3 times with 0.01 M phosphate buffered saline (PBS), incubated with 5% (w/v) bovine serum albumin (BSA) at 37°C for 30 minutes, and subsequently at 4°C overnight with antibody: PKM2 (1:50; dissolved by 0.01 M of PBS). Sections were rinsed in PBS three times, each time for 5 min, and incubated with a secondary antibody for 1 h at room temperature. For scoring of immunohistochemical staining of human specimens, both the proportion and the intensity of the positive staining were scored following published methods, with the proportion graded in 6 scales (0-5; ie, 0, none; 1, <1/100; 2, 1/100 to 1/10; 3, 1/10 to 1/3; 4, 1/3 to 2/3; and 5, more than 2/3), and the intensity graded in 4 scales (0-3; 0, none; 1, weak; 2, intermediate; and 3, strong). The total score from 0 (the minimum score) to 8 (the maximum score) was computed by combining the proportion and the intensity scores. The detailed score analysis was listed in **Table S1**.

### Cell viability assay

Cells (8×10^3^ cells/well) were plated evenly (96-well plate) and given different drugs for 72 hours. The next, 2mg/mL methyl thiazolyl tetrazolium solutions (50μL) were added to each well. Cell viability was measured the absorbance of each well at 490nm using a microplate reader (Biotek, SYNERGY HTX, Vermont, USA).

### Cologenic assay

Cells (8×10^3^ cells/well) were plated evenly (24-well plate) and given different drugs for 7 days. The next, the culture medium was discarded. Cells were carefully rinsed twice with PBS and fixed with 10% formalin for 2 hours. Appropriate amount of 0.1% crystal violet solutions was added to dye for 2 hours. Quantitative analysis was performed at 550 mm using a microplate reader (Biotek, SYNERGY HTX).

### Trans-well migration assay

Serum-free cell suspensions (2×10^4^ cells/well) were plated on the upper chamber and complete culture solutions were generally added to the lower chamber for 24 hours. Cells were fixed with 10% formaldehyde and stained with 0.1% crystal violet. For the cell migration assay in vitro, we first removed the stained cells on the upper surface. Then, the stained cells on the lower surface were photographed using microscopy (Leica, DFC450C; Wetzlar, Germany). The number of migrated cells in three random fields was counted.

### Cell apoptosis analysis

Cell apoptosis was detected with Annexin V FITC Apoptosis Kit (Becton, Dickinson and Company, New Jersey, USA). Treated cells were suspended in binding buffer and added 5 µl of Annexin V-FITC and 5 µl of PI for 20 minutes in the dark. The apoptotic cells were analyzed by FACS Calibur flow cytometer (Becton, Dickinson and Company, New Jersey, USA).

### Transfection of small interfering RNA (siRNA)

The PKM2 siRNAs were purchased from Ribobio Company (Shanghai, China). A2780, SKOV3 and OVCAR3 cells were transfected with Lipofectamine 2000 (Invitrogen, USA) and alongside siRNA (50 nM) in the absence of antibiotics or FBS for 6 hours. Then, the previous medium was replaced with the culture medium containing antibiotics and FBS and cells were carried out for 48 hours under normal conditions. Finally, specific silencing was confirmed by western blot. The sequences of the PKM2 siRNAs used in the study are shown in **Table S2**.

### Western blot analysis

Whole-cell lysate from each sample was loaded onto a 10% polyacrylamide gel for electrophoresis and transferred to PVDF membrane. After the primary antibody was allowed to incubate overnight at 4 °C, it was washed with PBS/0.1% Tween-20 and then incubated for an additional 1 hour using secondary antibody at room temperature. The proteins were visualized using Clarity Western ECL Substrate (Bio-Rad, California, USA) and the blot was imaged immediately on a ChemiDoc system (Tanon 4600, Shanghai, China). Quantification of protein expression was performed using Image J.

### Coimmunoprecipitation (Co-IP) assay

The following antibodies including PKM2, p-ATM antibodies were used in the Co-IP assays. In general, cell lysis was carried out using lysis buffer. After centrifugation, the supernatant was collected. The antibodies were added to lysates with protein A/G beads (Santa Cruz Biotechnology, Dallas, USA). Samples were incubated overnight. The beads were collected by centrifugation, the beads were then washed three times using IP buffer. Sample loading buffer (5×) was mixed with the beads and boiled for 10 min. The supernatant was used for western blot analysis.

### Cytosolic/nuclear fractionation

Separation of cytosolic and nuclear fractionation was performed using the Nuclear and Cytoplasmic Extraction Kit (CWBIOTECH, Beijing, China), according to the manufacturer's protocol. Cells 1 × 10^7^ were lysed in 1ml Nc-Buffer A incubate on the ice for 20 min. Then add 55 μL Nc-Buffer B incubate on the ice A for 1 min. Samples were centrifuged at 1,2000 rpm for 15 min at 4 ℃. The cytosolic supernatant fraction was collected on ice. The nuclear pellet was resuspend in Nc-Buffer C incubate on the ice for 40 min and centrifuged at 1,2000 rpm for 15 min at 4 ℃. Supernatant fractions were collected and analyzed, or stored at -80 ℃.

### Immunofluorescence (IF)

A2780, SKOV3 and OVCAR3 cells (2×10^5^ cells/well) were inoculated in 12-well plates with slides. Treated cells were fixed by 4% paraformaldehyde for 10 minutes. 0.5% Triton X‐100 was used to dissolve lipids and increased the permeability of cell membranes to antibodies. Then, blocking solution containing 5% BSA was utilized to block the slides for 30 minutes. A primary antibody for rabbit antihuman γH2AX using blocking solution at a ratio of 1:200 or a mouse antihuman BRCA1 diluted using blocking solution at a ratio of 1:50 was added. Then, cell cultures were mixed with the secondary antibodies diluted as indicated above. The slides were incubated in wet box at room temperature for 1 hour and stained with DAPI (Beyotime, Shanghai, China) for 10 minutes. Finally, the image was photographed using a fluorescence microscope (Leica, DFC450C; Wetzlar, Germany) in the dark.

### Mouse xenograft model and treatment

The experimental protocol was approved by Animal Welfare Ethics Committee and Laboratory Animal Management and Use Committee at Hunan Normal University (acceptance no. 2019029). Female athymic nude mice (BALB/c, 6-8 weeks old, nonfertile, 18-20g each) were purchased from Hunan SJA Laboratory Animal Co., Ltd (Changsha, China). Ola and Sk were dissolved in 5% DMSO, 20% PEG 400 and 5% Tween-80 and 70% distilled water. 1×10^7^ A2780 cells were subcutaneously injected into the right axilla of nude mice. Mice were randomly divided into 4 groups (n=6 per group) and given intraperitoneal injection when tumour grew to 100-200 cm^3^ (at 10 days after inoculation): (a) ctrl-treated group (once two day), 100μL; (b) Sk-treated group (2mg/kg, once two day), 100μL; (c) Ola-treated group (50 mg/kg, once a day), 100μL; and (d) combination-treated group [Ola (50 mg/kg, once a day) and Sk (2 mg/kg, once two day)]. The body weights and sizes of tumour were measured daily. Tumour volumes were calculated by the formula perpendicular (length×width^2^) / 2. Twelve days after treatment, the mice were killed, then tumours and major organs (liver and kidney) were removed for subsequent use in experiments.

### Histological analysis

Tumour tissues and the major organs (liver and kidney) were embedded in paraffin and then sectioned into 4-μm sections. PKM2, γH2AX, BRCA1 and Ki67 and hematoxylin-eosin (H&E) staining were conducted by the Department of Pathology, The Affiliated Zhuzhou Hospital of Xiangya School of Medicine. The slides were reviewed by a pathologist (Dr. Huang).

### Statistical analysis

The data are expressed as ± standard deviation. The two-sided Student's t-test was used for comparisons of two independent groups. All statistical analyses were performed within a 95% confidence interval, ***P< 0.001, **P < 0.01, *P < 0.05. The statistical analyses were performed via GraphPad Prism 6 and SPSS 16.0.

## Results

### PKM2 is highly expressed in ovarian carcinoma tissues

**Figure 1:**
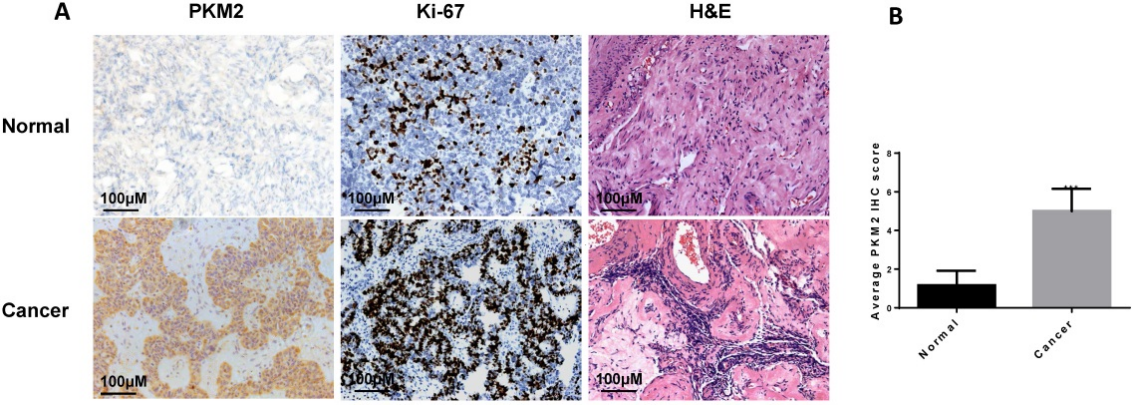
PKM2 is highly expressed in ovarian carcinoma tissues. A, the expression of PKM2 in tissues was evaluated via immunohistochemistry. Adjacent normal tissues (N=10) and cancer tissues (N=20) were immunohistochemically stained with anti-PKM2. H&E and Ki-67 were used to indicate whether there is a tumour; scale bar = 100 μm. B, PKM2 immunohistochemical scores of two groups (normal and tumour tissues). *** < 0.001, **P < 0.01, *P < 0.05, two-sided Student's t-test.

In this study, we found that PKM2 was highly expressed in ovarian carcinoma tissues than normal ovarian tissues by immunohistochemistry (**Fig. 1A-B**), consistent with observation in other publications 36, 37. The clinical characteristics of the sample are shown in **Table 1**.

### PKM2 plays a vital role in DNA damage by disrupting HR in ovarian cancer cells

**Figure 2:**
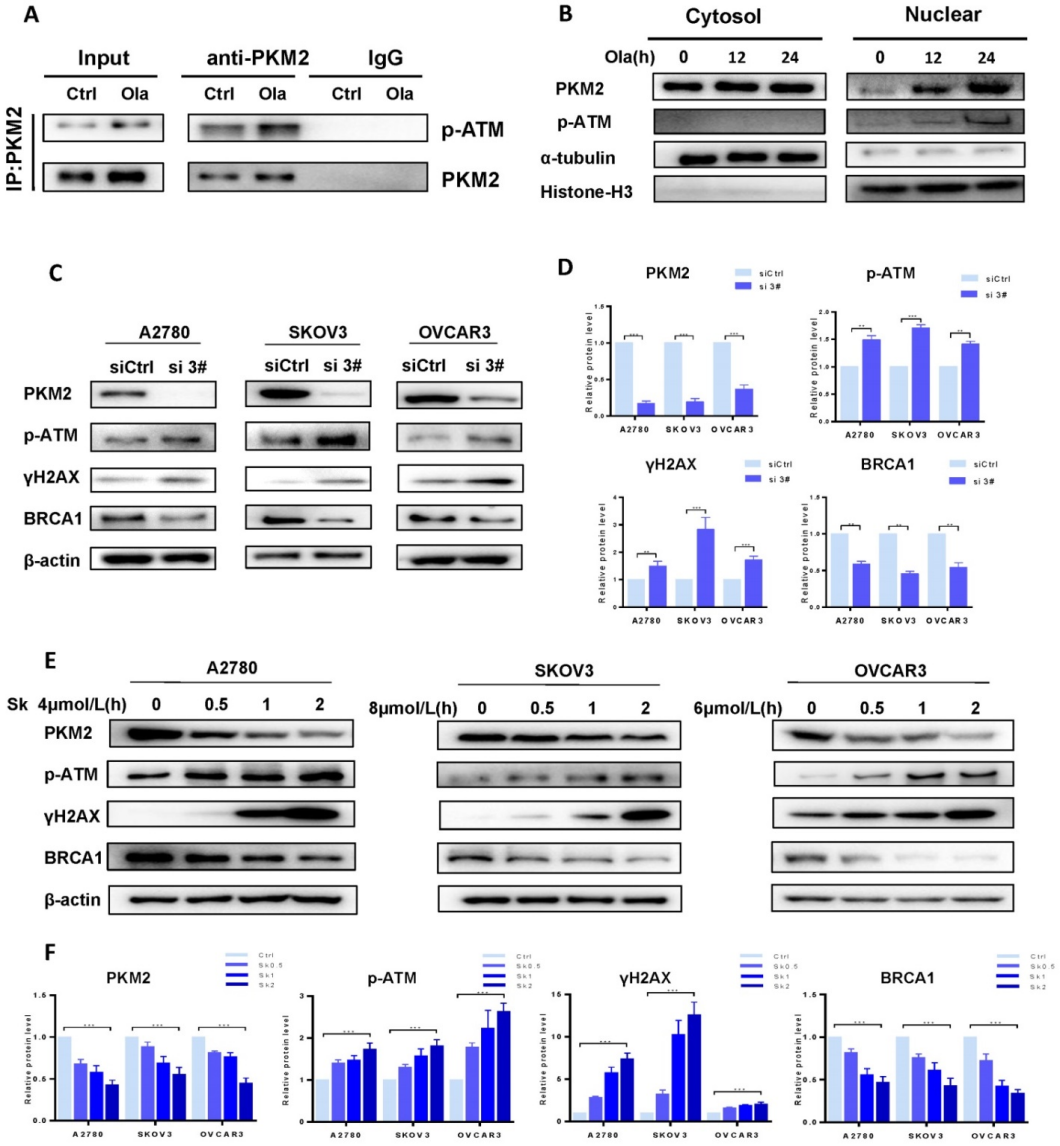
** PKM2 plays a vital role in DNA damage by disrupting HR in ovarian cancer cells.** A, Co-IP analysis of the interaction between endogenous p-ATM and PKM2 using anti-PKM2 or anti-p-ATM antibody in A2780 cells treated with or without Ola (10μM) for the 12 hours. IgG was a negative control. Input, 10% the whole cell lysate. B, A2780 cells were treated with Ola (10μM) for the 12 or 24 hours and cell lysates were collected at the indicated times and separated into nuclear and cytoplasmic fractions which were resolved by SDS-PAGE and probed with the indicated antibodies. C, The expressions of PKM2, p-ATM, γHA2X and BRCA1 after si 3# in A2780, SKOV3 and OVCAR3 cells. D, Quantification of protein expression using Image J. E, A2780, SKOV3 and OVCAR3 cells were treated with 4 μmol/L, 8μmol/L and 6 μmol/L Sk for 0-2 hours respectively, followed by Western blot analysis of cellular PKM2, γHA2X, p-ATM and BRCA1 expressions. F, Quantification of protein expression using Image J. Data represented the mean ± SD of three independent experiments. *** < 0.001, **P < 0.01, *P < 0.05, two-sided Student's t-test.

As well documented, p-ATM and γH2AX are well-known molecular makers of DNA damage and BRCA1 is a major HR repair support protein 38. To explore the potential functions of PKM2 in DNA damage-related events, we sought to determine the interaction between endogenous PKM2 and ATM. As shown in **Fig. 2A,** immunoprecipitating PKM2 by anti-PKM2 antibody pulled down p-ATM in either presence or absence of Ola. It suggested that PKM2 is linked with DNA damage pathway. Interestingly, Ola increases binding of p-ATM to PKM2. While the majority of PKM2 is located in cytoplasmic, PKM2 shuttles from the cytoplasmic to nucleus in the response to Ola treatment 39, as shown in western blot analysis of cytosolic and nuclear protein fractions (**Fig 2B**), consistent with previous observations 34.

To better understand the role of PKM2 on DNA damage, we sought to determine whether down-regulation of PKM2 by silencing could influence the status of HR deficiency in ovarian cancer cells. Ovarian cancer cells were transfected with 50 nM of the three PKM2 siRNA sequences for 72 hours. Results of the western blotting analysis of whole cell lysates showed the more robust reduction in PKM2 protein expression in the ovarian cancer cells transfected with si 2# and si 3# compared with PKM2 siRNA 1# sequence, and si 3# had the highest silencing efficiency (**Fig. S1**). Therefore, si 3# was chosen for the following series of studies, and si 2# was used to verify results of some experiments. At first, the expression levels of p-ATM, γH2AX and BRCA1 were compared between si 3#-treated and siCtrl-treated in A2780, SKOV3 and OVCAR3 by western blotting. The levels of p-ATM and γH2AX are increased and the levels of BRCA1 are decreased in si 3#-treated cells (**Fig. 2C-D**).

The approach of targeting PKM2 via siRNA is limited in current clinical utilization since clinical delivery of siRNA faces a challenge in treating cancer patients. Therefore, we applied the second approach for targeting PKM2, which is a PKM2 inhibitor Sk, to determine the alteration of ATM/γH2AX and BRCA1 levels. Cells were treated with DMSO or different doses of Sk for 0-2 hours. We examine changes of PKM2, p-ATM, γH2AX and BRCA1 protein levels in presence and absence of Sk treatment. Sk treatment resulted in PKM2 expression reduced by 60% relative to the DMSO control cells and the results demonstrated that Sk treatment markedly upregulated p-ATM and γH2AX protein levels and reduced BRCA1 protein levels in a time-dependent manner (**Fig. 2E-F**). Thus, all above results indicate that inhibition of PKM2 is associated with inducing DNA damage and disruption of HR in ovarian cancer cells, which would potentially render them susceptible to PARPi. In order to further elucidate the relationship between PKM2 and DNA damage, we explored the effect of overexpression of PKM2 on DNA damage, and found that overexpression of PKM2 can cause down-regulation of p-ATM. Next, we conducted a rescue test of PKM2. Statistical data showed compared with silencing alone, rescue of PKM2 by overexpression reversed the DNA damage-related events caused by silencing PKM2 (**Fig. S2**). Therefore, it preliminarily verified that PKM2 plays a vital role in DNA damage.

### Downregulation of PKM2 enhances ola-induced DNA damage-related events

**Figure 3:**
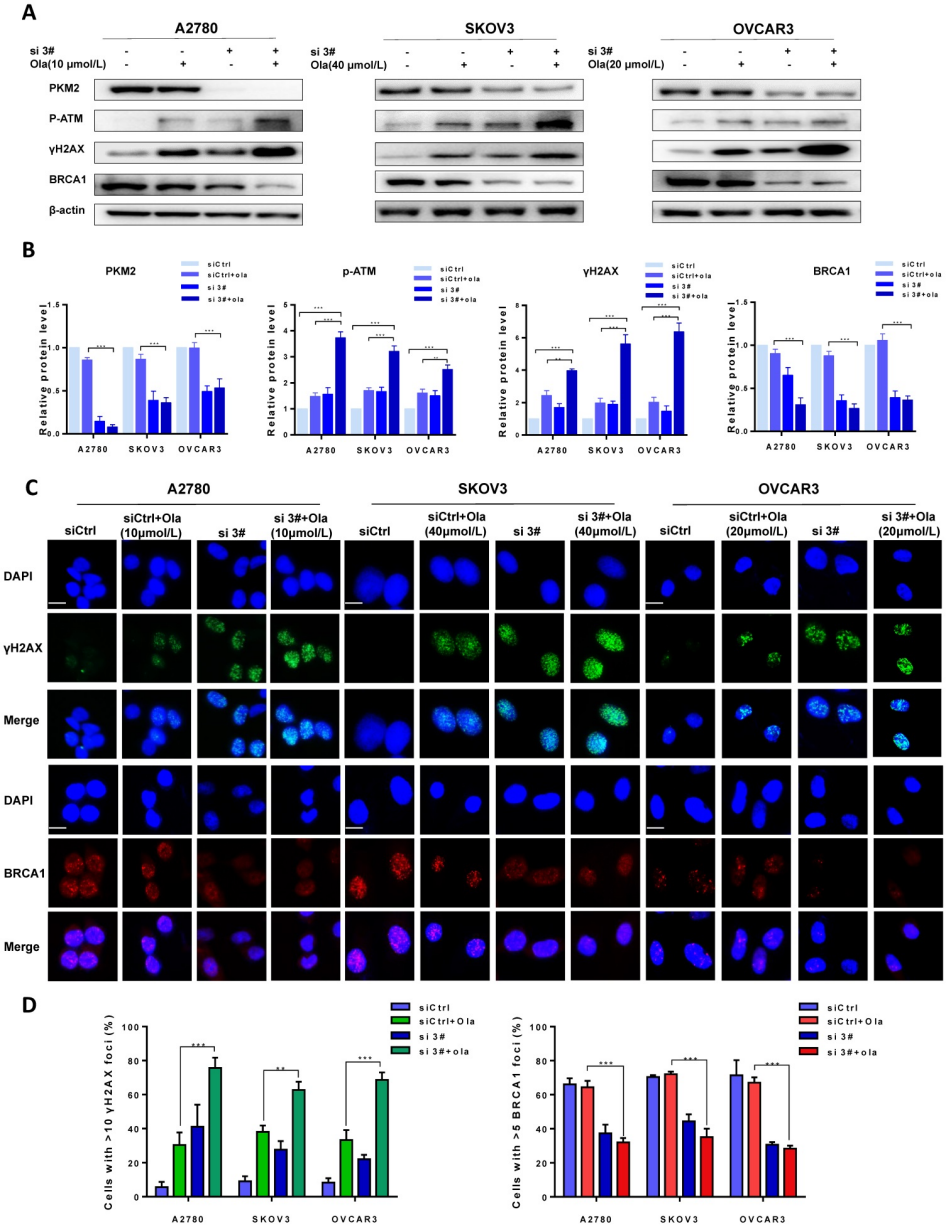
Downregulation of PKM2 enhances Ola-induced DNA damage-related events. A, A2780, SKOV3 and OVCAR3 cells were transfected with control siRNA or PKM2 siRNA and then treated with Ola for 12 hours respectively, followed by Western blot analysis of cellular PKM2, γHA2X, p-ATM and BRCA1 expressions. B, Quantification of protein expression using Image J. C-D, the expression of γHA2X and BRCA1 using IF analysis after si 3# combined with 10 μmol/L, 40μmol/L and 20μmol/L Ola treatment. Cells were assessed after treatment with si 3# alone or combined with Ola 12 hours; scale bar = 20 μm. Results are the mean ± SD of three independent experiments. *P < 0.05, **P < 0.01, *** < 0.001, two-sided Student's t-test.

The protein levels of PKM2, p-ATM, γH2AX and BRCA1 were compared between si 3# pre-treated and siCtrl pre-treated in presence and absence of Ola treatment to determine the effect of PKM2 downregulation on DNA damage-related proteins. As shown in **Fig. 3A-B**, Ola activated p-ATM and γH2AX but had little effect on BRCA1, consistent with previous observations.^40^ Interestingly, silencing PKM2 combined with Ola treatment further activated the protein levels of p-ATM and increased the expression of γH2AX while BRCA1 expression decreased slightly.

To further confirm amplification effect of PKM2 downregulation on Ola-induced DNA damage-related events, immunofluorescence staining was applied to determine the foci formation of γH2AX and BRCA1 in different treatment cells. The foci formation was measured by determining the percentage of cells with >10 γH2AX foci and >5 BRCA1 foci 41. We tested whether pre-treatment with si 3# affected γH2AX and BRCA1 foci formation. As shown in **Fig. 3C**, the γH2AX foci formation significantly increased after silencing PKM2. In addition, Ola treatment further increased γH2AX foci formation in silencing PKM2 ovarian cancer cells. The next, we measured the critical HR protein BRCA1 foci formation. It was found that the percentage of BRCA1 foci formation significantly decreased in si 3#-treated cells compared to Ola-treated and siCtrl-treated cells. These results indicated that pre-treatment with si 3# significantly enhanced Ola-induced DNA damage-related events (**Fig. 3D**).

**Figure 4:**
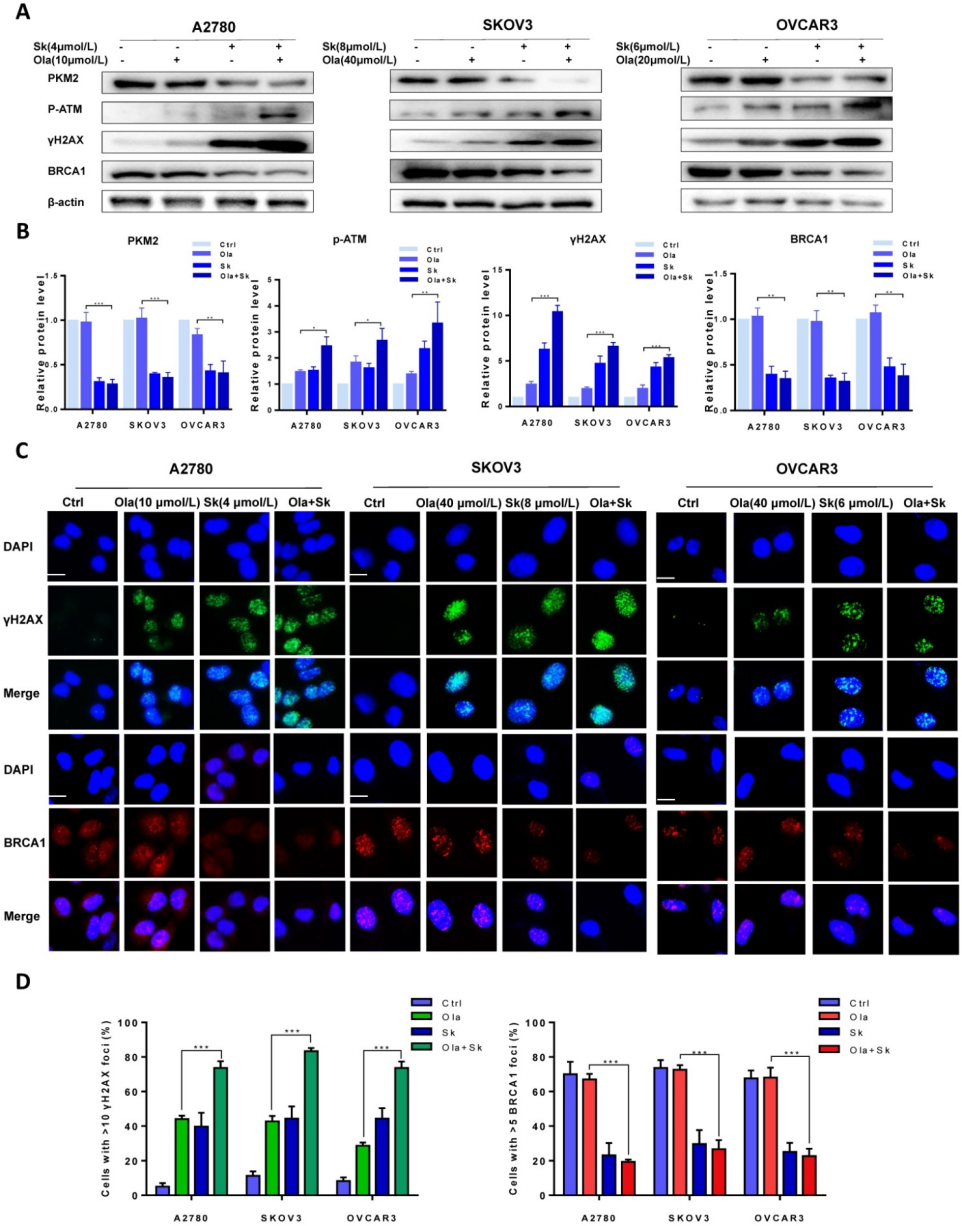
Sk enhances Ola-induced DNA damage-related events. A, Western blot analysis for PKM2, γHA2X and BRCA1 in A2780, SKOV3 and OVCAR3 cells after Sk combined with Ola treatment. Cells were assessed after treatment with Ola 12 hours alone or combined with Sk 2 hours. B, Quantification of protein expression using Image J. C-D, the expression of γHA2X and BRCA1 using IF analysis after Sk combined with Ola treatment; scale bar = 20 μm. Results are the mean ± SD of three independent experiments. *P < 0.05, **P < 0.01, *** < 0.001, two-sided Student's t-test.

Further to determine whether that downregulation of PKM2 enhanced Ola-DNA lesions and attenuated HR repair efficiency, cells were pre-treated with PKM2 inhibitor Sk or DMSO and then exposed to Ola for 12 hours. Similarly, the protein levels of PKM2, phospho-ATM and γH2AX and BRCA1 were compared between Sk pre-treated and DMSO pre-treated in presence and absence of Ola treatment. Our results clearly show that inhibition of PKM2 by Sk amplified Ola-induced γH2AX upregulation and phospho-ATM activation and BRCA1 downregulation (**Fig. 4A-B**). In addition, PKM2 inhibitor Sk also increased Ola-induced γH2AX foci formation and downregulated BRCA1 foci formation in all three ovarian cancer cell lines (**Fig. 4C-D**). We found that PKM2 downregulation by Sk changes the recruitment of proteins involved in DNA damage repair by HR. These solid facts documented that PKM2 downregulation sensitizes ovarian cancer cells to Ola treatment.

### Inhibition of PKM2 amplifies inhibitory activity of Ola on ovarian cancer cell growth

**Figure 5:**
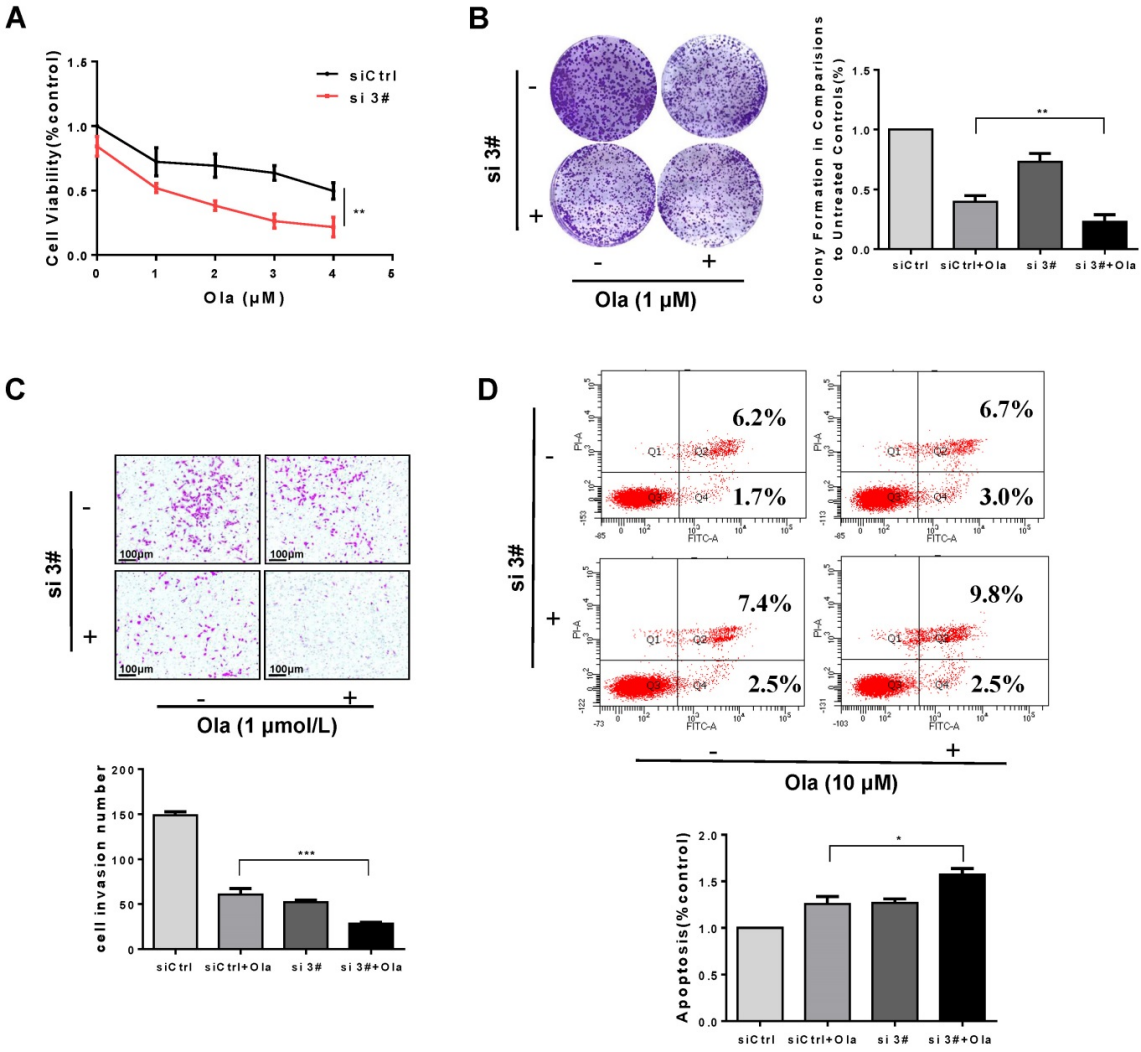
Silencing PKM2 amplifies inhibitory activity of Ola on ovarian cancer cell growth. A, Different concentrations of Ola alone or combined with si 3# treatment using cell viability assay. B, Colony formation assay results of si 3# combined with Ola treatment on A2780 with 7 days' treatment. C, Transwell assay results of si 3# combined with Ola treatment on A2780; scale bar = 100 μm. D, Apoptosis experiment was assessed with 12 hours Ola alone or combined with si 3# treatment on A2780. Data represented the mean ± SD of three independent experiments. *** < 0.001, **P < 0.01, *P < 0.05, two-sided Student's t-test.

Targeting PKM2 at protein levels in cancer cells results in defective HR. Destruction of HR has been shown to be sensitive to PARPi. Therefore, we further explore whether inhibition of PKM2 could enhance antitumor efficacy of PARPi Ola. As shown in **Fig. 5** and **Fig. S3**, ovarian cancer cells were treated with si 3#, si 2# or siCtrl and then exposed to Ola. The inhibitory effect of Ola on ovarian cancer cells was detected by cell viability assay (**Fig. 5A** and **Fig. S3A**), and cologenic assay **(Fig. 5B** and **Fig S3B**). The results showed that si 2# or si 3# both enhanced the anti-tumor activity of Ola. Trans-well assay (**Fig. 5C** and **Fig. S3D**) also proved that the combination of si 3# and Ola further inhibited cell migration. In addition, we analyzed the effect of si 3# on Ola-treated cell apoptosis. As shown in **Fig. 5D,** silencing PKM2 or inhibiting PARP by Ola treatment increased apoptosis levels in A2780 cells. More importantly, silencing PKM2 in combination with Ola treatment further increased apoptosis ratio.

### Combination of PKM2 inhibitor Sk with Ola exerts synergistic effect on anti-ovarian cancer *in vitro*

**Figure 6:**
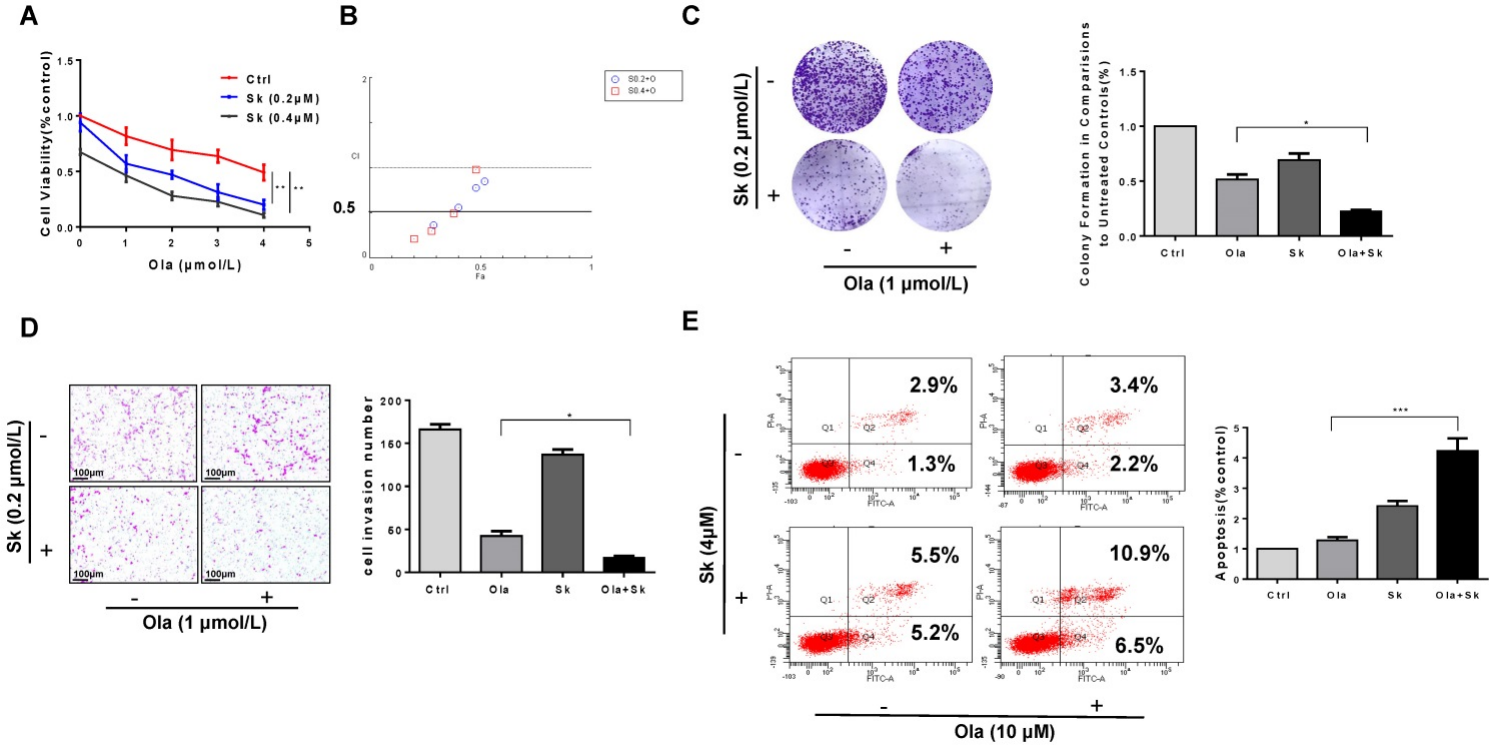
Combination of PKM2 inhibitor Sk with Ola exerts synergistic effect on anti-ovarian cancer *in vitro*. A, Sk combined with Ola inhibited A2780 cells proliferation synergistically. B, the combination index (CI) assessing synergy between the two drugs was calculated. CI = 1 denotes additivity; CI > 1, antagonism; CI < 1, synergism. C, Ola alone or combined with Sk treatment on A2780 cells using colony formation assay. D, Combination of Sk with Ola inhibited cell invasion of A2780 cells by transwell assays; scale bar = 100 μm. E, Apoptosis experiment was assessed with 12 hours Ola alone or combined with Sk for 2 hours' treatment on A2780 cells. Data represented the mean ± SD of three independent experiments. *** < 0.001, **P < 0.01, *P < 0.05, two-sided Student's t-test.

As shown in **Fig. 6A**, the viability of A2780, SKOV3 and OVCAR3 cells treated with combination of Sk and Ola was largely reduced compared to Ola or Sk treatment alone. Using the CompuSyn software, Combination Index (CI) of Ola and Sk was obtained, ranging from 0.23 to 0.97 for A2780 cells (**Fig. 6B**). These results indicated a strong synergy for these combinations. Consistently, the combination significantly inhibited clone formation and migration, much greater than treated with Ola alone (**Fig. 6C-D**). In SKOV3 and OVCAR3 cells, we also performed the same experiments and observed similar results (**Fig. S4**). Furthermore, the results of apoptosis (**Fig. 6E**) showed that treatment of A2780 cells with Sk, Ola or their combination for 12h significantly induced apoptosis, consistent with the results of cell viability assay.

### Treatment of Sk in xenograft tumor mice augmented anti-tumor effect of Ola

**Figure 7:**
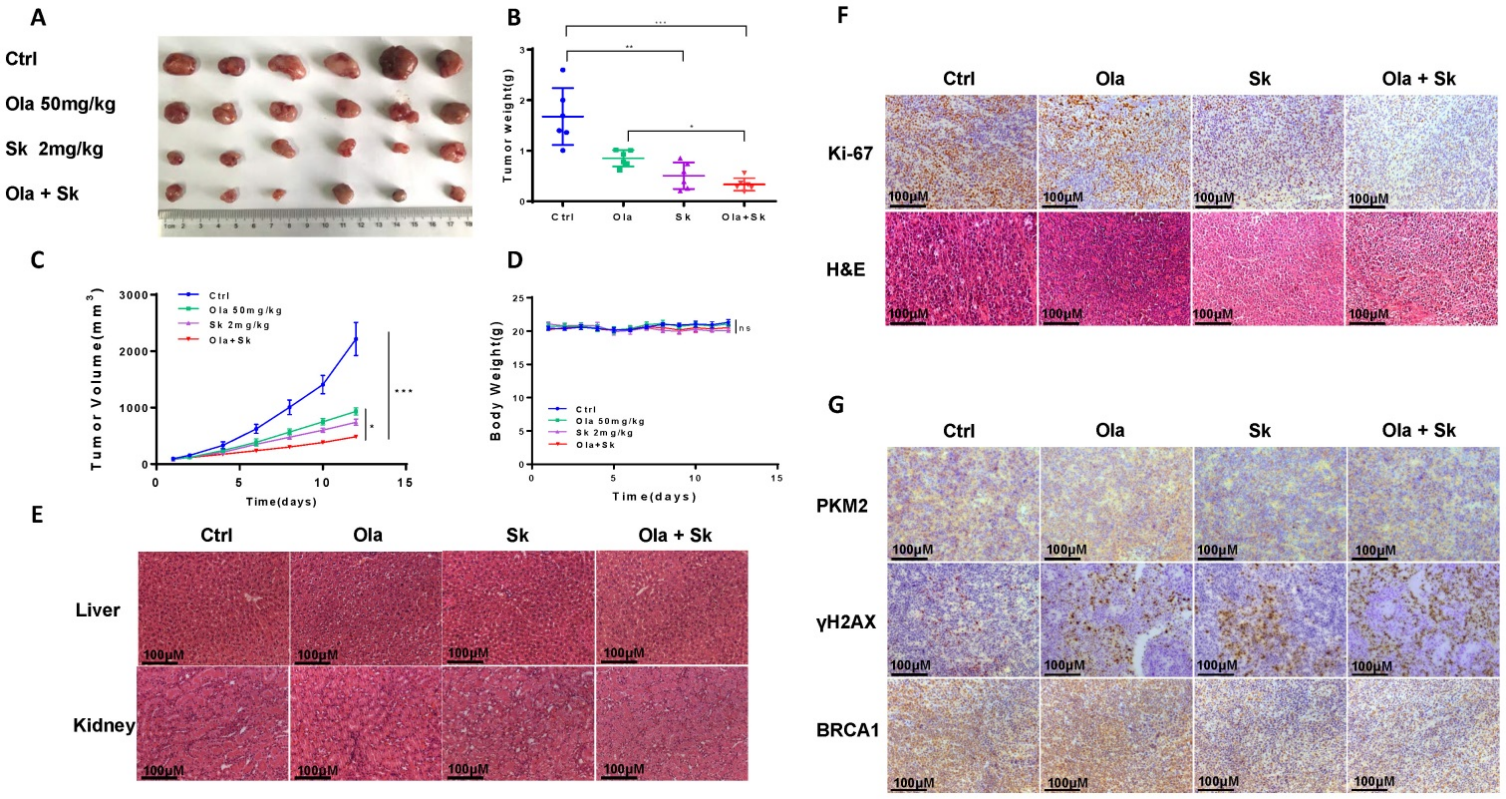
Treatment of Sk in xenograft tumour mice augmented anti-tumour effect of Ola. A, the picture of xenograft tumors was taken after the treatment.the combination-treated group (Ola and Sk B, Nude mice tumour weights after 12 days of administration. C, Tumour volumes were calculated every two days. D, Body weights were measured every day. E, Liver and kidney tissue morphology were observed via H&E staining; scale bar = 100 μm. F, the expression of Ki-67 and H&E staining in tumour tissues; scale bar = 100 μm. G, The expression of PKM2, γH2AX and BRCA1 of tumour tissues from nude mice of 4 groups using immunohistochemistry; scale bar = 100 μm. *P < 0.05, **P < 0.01, *** < 0.001, one-way ANOVA.

The anti-tumour effect of Ola was evaluated after combination with Sk using xenograft tumor mice. Nude mice injected subcutaneously with A2780 cells were treated with Ola alone and in combination with Sk for 12 days. As shown in **Fig. 7A-C**, compared with ctrl-treated group, 50mg/kg Ola-treated group alone diminished the tumor volume. Surely, the effect is not statistically significant while 2mg/kg Sk-treated group alone exhibited mild effect. Tumor growth was efficiently inhibited by combination of Ola and Sk and the volume of the tumors treated with combination was one-five of the volume of ctrl-treated group at Day 12 (p<0.001). No obvious difference of body weight of mice between Ola-treated group and ctrl-treated group was observed while body weight of mice in Sk-treated group tended to decrease around 5-7% after 8-12 days of treatment, indicating the presence of mild toxicity of Sk (**Fig. 7D**). In contrast, body weight of combination-treated mice is higher than that of Sk-treated group, closer to ctrl-treated group. This result suggested that Ola could effectively attenuate weight loss caused by Sk. Liver and kidney of all treated groups are collected at the end of the experiment. H&E staining showed that, compared with the ctrl-treated group, no obvious pathological alterations are observed in all drug-treated groups (**Fig. 7E**). The immunohistochemistry analysis of Ki-67 demonstrated that the number of positive tumor cells in combination-treated group was significantly decreased compared with ctrl-treated group (**Fig. 7F**). In support of the mechanism discovered in this study, immunohistochemistry results demonstrated that Sk-treated and combination-treated groups decreased the expression of PKM2 and BRCA1. The expression of γH2AX is the highest in the combination-treated group (**Fig. 7G**). Collectively, these results demonstrated that Sk enhanced anti-tumour activity of Ola against ovarian tumours *in vivo*. Thus, PKM2 inhibitor might have a great potential in combination with Ola for clinically treating ovarian cancer.

## Discussion

In this study, we demonstrated for the first time that the expression of PKM2 was inversely related to the efficacy of Ola against ovarian tumors. Our results indicate that downregulation of PKM2 with either siRNA or PKM2 inhibitor Sk reduced expression of HR components and increased DNA damage. Furthermore, overexpression of PKM2 caused down-regulation of p-ATM. Rescue of PKM2 after silencing reversed the DNA damage-related events caused by silencing PKM2. It showed that PKM2 is closely related to DNA damage. Otherwise, either PKM2 siRNA or Sk combined with Ola showed synergistic anti-tumor activity in ovarian cancer cell lines. Finally, we validated the effectiveness of Sk with Ola in a xenograft animal model. These results suggest a potential therapeutic strategy for the use PKM2 inhibitor in combination with PARPi to women diagnosed with BRCA1/2 wild type ovarian cancer.

Constant exposure to genotoxic insults in cancer cells cause severe damage to the DNA structure due to a plethora of different modifications, highlighting the importance of HR in the repair process 42. PKM2 is known to catalyze the last step of glycolysis to sustain the bioenergetics and biosynthetic demands of cell proliferation 25. Reports suggest that PKM2 deletion facilitates apoptosis induced by DNA damaging agents and decreases proliferation of tumor cells 22, 32-34. Thus, PKM2 interacts with the DNA damage response and repair system although detailed molecular mechanisms are largely unknown. Our results advanced the current understanding by showing that downregulating of PKM2 affected the expression of the HR-related proteins ATM, γH2AX and BRCA1 in ovarian cancer cells. Ola does not affect BRCA1 expression while PKM2 silencing does. It is well known that PKM2 is mostly located in the cytoplasm, but it can also shuttle between cytoplasm and nucleus in the presence of certain stimulus 43. Our results confirmed that Ola promoted the nuclear accumulation of PKM2, consistent with previous study 34. PARPi kills tumor cells defective in HR-based repair but not in HR-proficient counterparts 44, 45. The most widely accepted mechanism of PARPi resistance is the restoration of BRCA function or HR repair via secondary mutations 46, 47. Thus, the reduction of BRCA function or decrease in HR repair is critical to improve anti-tumor effectiveness of PARPi. Several approaches have been evaluated preclinical such as combinations of PARPi with UCHL3 48 inhibitors or CDK4/6 49 inhibitors or PI3K/AKT inhibitors 50, 51 or suppression of cyclin D1 52, which enhance cytotoxicity of PARPi through the disruption of HR repair. Furthermore, our present study provides evidence for a better understanding of improvement of the sensitivity of ovarian cancer cells to Ola. Downregulation of PKM2 by either PKM2 siRNA or PKM2 inhibitor Sk treatment amplified Ola-induced γH2AX and p-ATM expression. Therefore, downregulating PKM2 protein level resulted in defective HR pathway in BRCA1 wild type ovarian cancer cells. All results of cell viability, clone formation and Tran swell assays and apoptosis experiments confirmed that the sensitivity of three ovarian cancer cell lines to Ola increased profoundly after knocking down PKM2.

Many studies have proven that combining Sk with a variety of anti-tumor drugs synergistically suppresses tumor growth and overcomes drug resistance 53-55. Zhou found that Sk, a specific inhibitor of PKM2, induced upregulation of RIP1 and RIP3, necrotomic formation and DNA DSBs 56. As mentioned previously, the present study is the first to reveal that Sk enhanced the effectiveness of Ola on inhibiting cancer cell growth. However, the underlying mechanisms of Ola combined with Sk have not been studied. Exploration of the detailed molecular mechanisms demonstrated that DNA damage related biomarkers γH2AX and p-ATM upregulated by Ola treatment were further enhanced and BRCA1 protein levels were further reduced when PKM2 was specially blocked by Sk. The induction of DNA double-strand breaks from increased intracellular reactive oxygen species caused by Sk itself could be the additional mechanism as reported previously 56. Furthermore, we observed similar combination effects of Sk and Ola via *in vivo* results, demonstrating convincingly that PKM2 inhibition by Sk further suppressed Ola-treated xenograft tumor growth without observable toxicity. This combination presents a new opportunity for employing PARPi therapy in aggressive ovarian cancers with active HR pathways. In conclusion, we established a foundation for using PKM2 inhibitor to sensitize ovarian cancer to Ola treatment, which may provide a novel targeted treatment strategy for advanced ovarian cancer.

## Supplementary Material

Supplementary methods and figures.

## Figures and Tables

**Table 1:** Clinical characteristics of the patients.

Features	Number of case (%)
**Age**	
≤55	12 (60%)
>55	8 (40%)
**Menopausal status**	
Pre-menopausal	6 (30%)
Post-menopausal	14 (70%)
**FIGO staging**	
Low stage (Ⅰ, Ⅱ)	5 (25%)
High stage (Ⅲ, Ⅳ)	15 (75%)
**Histology**	
Serous	17 (85%)
Other	3 (15%)
**Differentiation**	
High grade	17 (85%)
Low grade	3 (15%)
**Surgical outcome**	
Optimal (residual < 1 cm) debulking	13 (65%)
Nonoptimal debulking	4 (20%)
Not done	3 (15%)

## Abbreviations

PARPi: Poly (ADP-ribose) polymerase inhibitors
HR: homologous recombination
PKM2: Pyruvate kinase M2
Sk: shikonin
Ola: olaparib
p-ATM: phospho-ATM
BER: Base excision repair
SSB: single-strand breaks
DSB: DNA double-strand break
PK: Pyruvate kinase
DMSO: dimethyl sulfoxide
CST: Cell Signaling Technology
PBS: phosphate buffered saline
BSA: bovine serum albumin
Co-IP: Coimmunoprecipitation
H&E: hematoxylin-eosin
CI: The combination index
