# Supplementary material for: Inhibition of PKM2 Enhances Sensitivity of Olaparib to Ovarian Cancer Cells and Induces DNA Damage
Source: Int J Biol Sci. 2022 Jan 24;18(4):1555–68. doi: 10.7150/ijbs.62947 (PMC8898351; doi:10.7150/ijbs.62947)
Supplement: Supplementary file 1 — Supplementary methods and figures. [file ijbsv18p1555s1.pdf]

Supporting Information

Inhibition of PKM2 Enhances Sensitivity of Olaparib to Ovarian Cancer Cells and Induces DNA Damage

Sichun Zhou[1#], Duo Li[1#], Di Xiao[1], Tianyu Wu[2], Xin Hu[1], Yu Zhang[2], Jun Deng[1], Jing Long[2], Simeng Xu[1], Jingtao Wu[1], Gaofeng Li[2], Mei Peng[2] and Xiaoping Yang[1]*

#These authors contributed equally to this work.

*Correspondence should be addressed to Xiaoping Yang,

E-mail: xiaoping.yang@hunnu.edu.cn.

Includes:

1. Supplementary Materials and methods
2. Supplementary tables (2 tables)
3. Supplementary figures (4 Figures)

Supplementary Materials and methods

Chemicals and antibodies

The chemicals including Ola and Sk were purchased from Targetmol (Shanghai, China). Both Ola and Sk were dissolved in dimethyl sulfoxide (DMSO), dissolution with a 20 mM stock solution. Primary antibodies against β-Actin (#8457), PKM2 (#4053) were provided by Cell Signaling Technology (Boston, USA). p-ATM (phosphoSer1981; ab81292) was provided by Abcam (Cambridge, UK). The secondary antibodies anti-rabbit IgG (#7074) and anti-mouse IgG (#7076) were provided by CST. Lipo6000™ (C0526) was provided by Beyotime (Boston, USA). Plasmids pCMV3-PKM-GFP Spark and plasmids pCMV3-GFPSpark were provided by Sino Biological Inc. (Beijing, China).

Overexpression of PKM2

According to the instructions of transfection reagent, SKOV3 cells (2*10^5 cells/well) were transfected with plasmid pCMV3-PKM-GFPSpark vector or the control vector using transfection reagent Lipo6000™ for 6 hours. After transfection, the cells were cultured in complete medium for 48 hours. Finally, the samples were collected for detection of western blot.

Rescue experiment

Transfection of plasmid or siRNA was performed using Lipo6000™. According to the instructions of transfection reagent, SKOV3 cells ($2*10^5$ cells/well) were transfected with PKM2 siRNA 3# for 6 hours, then cells were cultured in complete medium for 24 hours. Above cells were continued transfected with plasmids pCMV3-PKM-GFPSpark vector or pCMV3 –GFP Spark vector for 6 hours, the samples were collected at 48 hours for western blot after transfection of plasmid.

Cell viability assay

Cells (8×10^3 cells/well) were plated evenly (96-well plate) and given different drugs for 72 hours. The next, 2 mg/mL methyl thiazolyl tetrazolium solutions (50 μL) were added to each well. After 4h, DMSO (150 μl; Sigma) was added to dissolve formazan crystals, and absorbance was measured using a microplate reader (Synergy HTX; Biotek, VT, USA) at 490 nm.

Cologenic assay

Cells (8×10^3 cells/well) were plated evenly (24-well plate) and given different drugs for 7 days. The next, the culture medium was discarded. Cells were carefully rinsed twice with PBS and fixed with 10% formalin for 2 hours. Appropriate amount of 0.1% crystal violet solutions was added to dye for 2 hours. Quantitative analysis was performed at 550 mm using a microplate reader.

Transwell migration assay

Serum-free cell suspensions (2×10^4 cells/well) were plated on the upper chamber and complete culture solutions were generally added to the lower chamber for 24 hours. Cells were fixed with 10% formaldehyde and stained with 0.1% crystal violet. For the cell migration assay in vitro, we removed the stained cells on the upper surface first. Then, the stained cells on the lower surface were photographed using microscopy. The number of migrated cells in three random fields was counted.

Supplementary table

Table S1. The detailed score analysis for immunohistochemical staining of human specimens.

**Scoring**		**The proportion of the positive staining**			
		None	Weak	Intermediate	Strong
**The intensity of the positive staining**	None	0	1	2	3
	< 1%	1	2	3	4
	1% to 10%	2	3	4	5
	10% to 33%	3	4	5	6
	33% to 67%	4	5	6	7
	> 67%	5	6	7	8

Table S2. The sequences of the PKM2 siRNAs.

siRNAs	Sequences
si 1#	CCAACACCAUGCGUGUUGU
si 2#	GCCAUAAUCGUCCUCACCA
si 3#	CCAUAAUCGUCCUCACCAAUU

Supplementary Figures

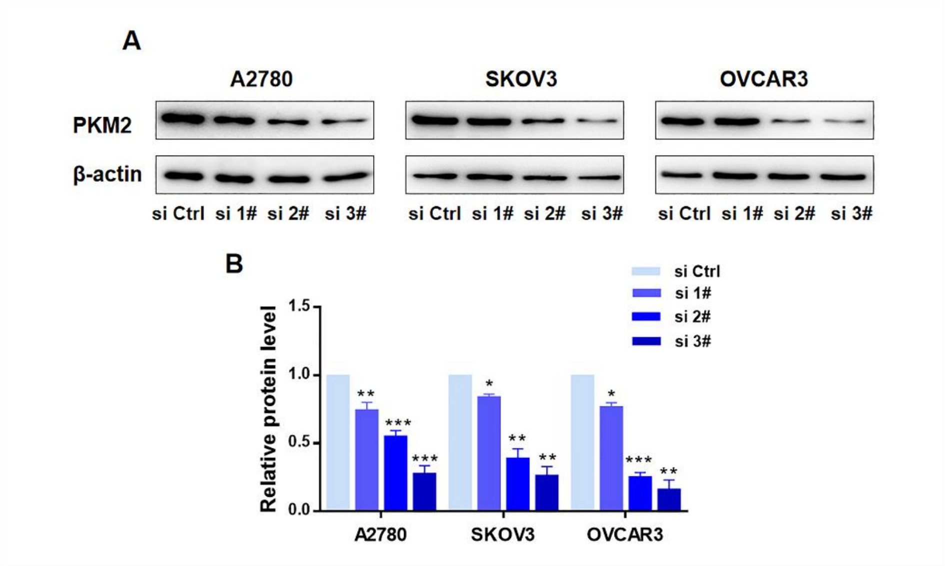

Figure S1, Effect of siRNA transfection on PKM2 expression in three types of ovarian cancer cells: A2780, SKOV3, and OVCAR3 cells. A, Expression level of PKM2 after transfected with PKM2 siRNAs (50 nM concentration for 72 h). β-actin was used as an internal loading control. siCtrl: si control, SiPKM2: si 1#, si 2#, si 3#. B, The statistical quantification of the western blot bands of PKM2. One-way ANOVA was used to compare the means of different groups. Differences between the means were considered to be significant at $P<0.05$ by using Tukey multiple comparison tests; $^{*}P < 0.05$, $^{**}P < 0.01$, and $^{***}P < 0.001$ compared with the siCtrl-transfected cells.

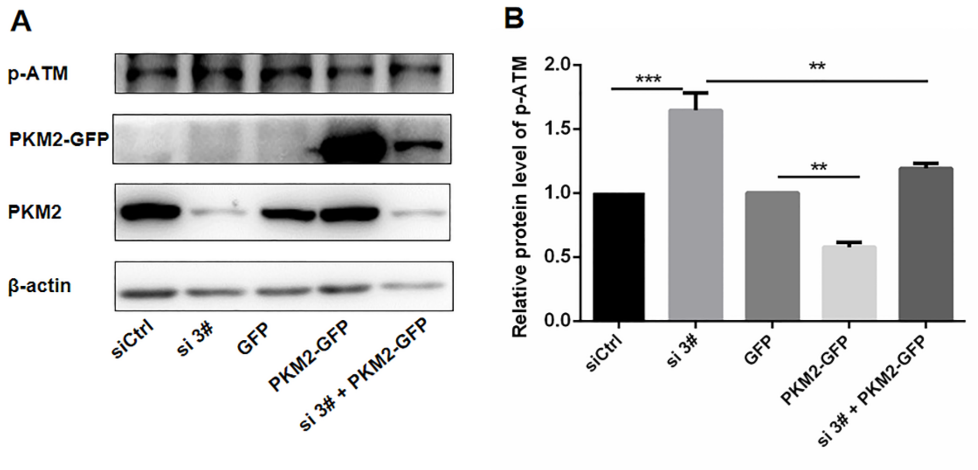

Figure S2, The effects of rescue experiment of PKM2 on the protein level of p-ATM in ovarian cancer SKOV3 cells. A, Protein levels of p-ATM were detected by western blot. β-actin was used as an internal loading control. SiCtrl: control; Si 3#: siRNA of PKM2; GFP: plasmids pCMV3–GFP Spark, as a control plasmid of PKM2-GFP; PKM2-GFP: plasmid CMV3-PKM-GFP Spark. B, The statistical quantification of the western blot bands of p-ATM. One-way ANOVA was used to compare the means of different groups. Differences between the means were considered to be significant at $P<0.05$ by using Tukey multiple comparison tests; *$P < 0.05$, **$P < 0.01$, and ***$P < 0.001$ compared with the si Ctrl-transfected cells, or plasmids pCMV3 –GFP Spark vector -transfected cells.

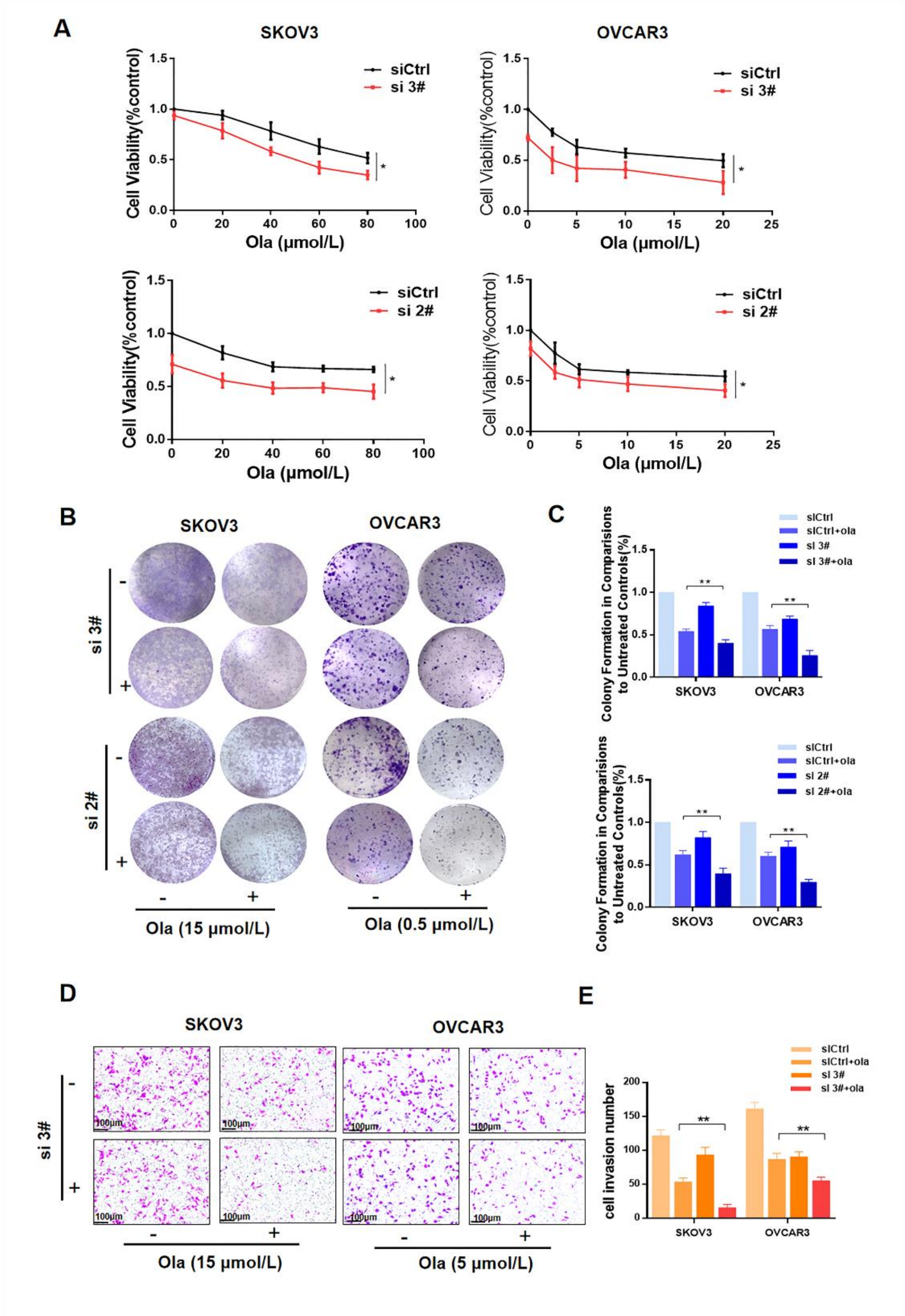

Figure S3, Silencing PKM2 amplifies inhibitory activity of olaparib on SKOV3 and OVCAR3 cells.

A, Different concentrations of olaparib alone or combined with siPKM2 (si 2# or si 3#) treatment using cell viability assay. B-C, Colony formation assay results of si 2# or si 3# combined with olaparib treatment with 7-days treatment. D-E, Transwell assay results of si 3# combined with olaparib treatment, scar bar = 100 μM. Data represented

the mean ± SD of three independent experiments. ***$P < 0.001$, **$P < 0.01$, *$P < 0.05$, two-sided Student's t-test.

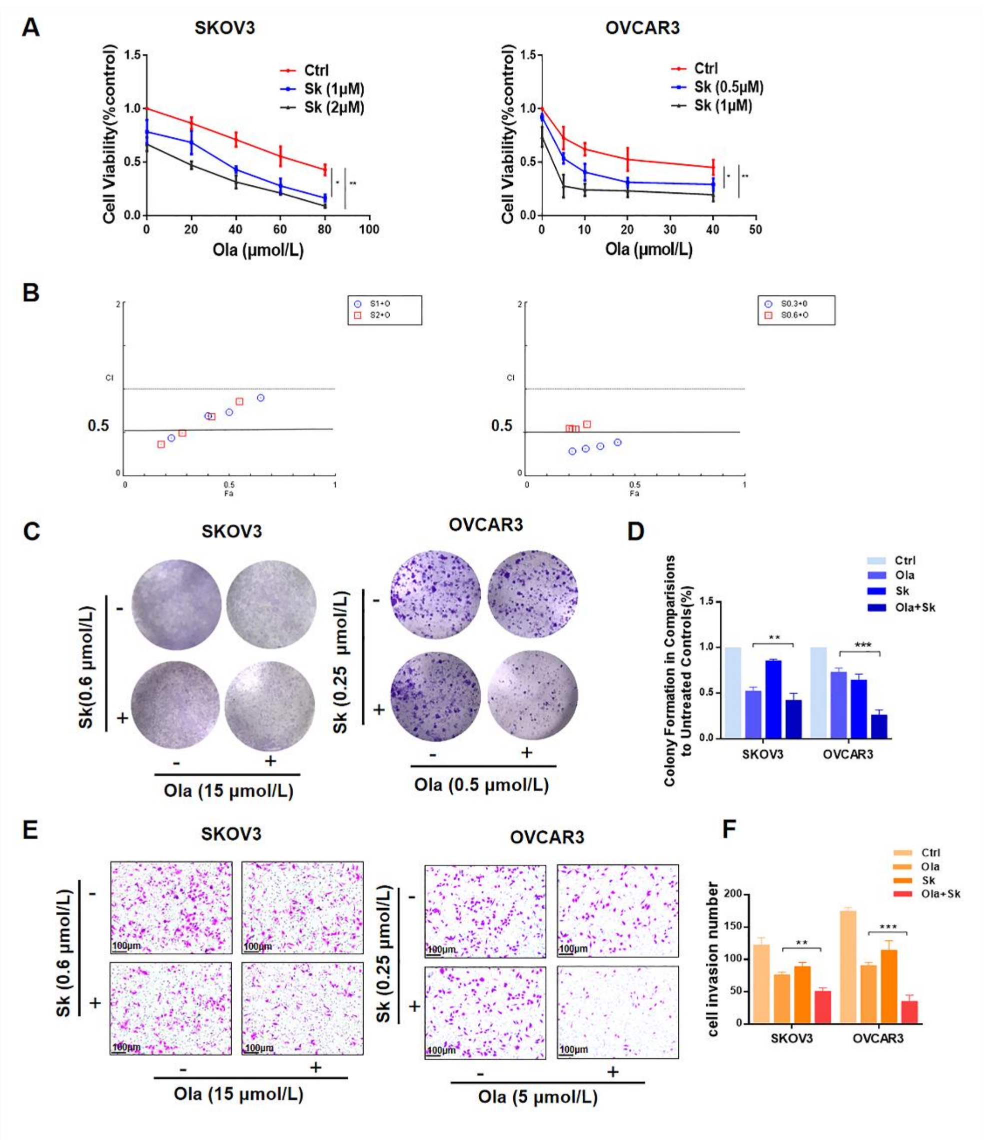

Figure S4, Combination of PKM2 inhibitor shikonin with olaparib exerts synergistic effect on SKOV3 and OVCAR3 cells *in vitro*.

A, Shikonin combined with olaparib inhibited SKOV3 and OVCAR3 cells proliferation synergistically. B, Combination index (CI) assessing synergy between the two drugs was calculated. CI = 1 denotes additivity; CI > 1, antagonism; CI < 1, synergism. C-D, Olaparib alone or combined with shikonin treatment on SKOV3 and OVCAR-3 cells using colony formation assay, scar bar = 100 μM. E-F, Combination of shikonin with olaparib inhibited cell invasion of SKOV3 and OVCAR3 cells by transwell assays. Data represented the mean ± SD of three independent experiments. ***P < 0.001, **P < 0.01, *P < 0.05, two-sided Student's t-test.
